# Construction of predictive model of interstitial fibrosis and tubular atrophy after kidney transplantation with machine learning algorithms

**DOI:** 10.3389/fgene.2023.1276963

**Published:** 2023-11-01

**Authors:** Yu Yin, Congcong Chen, Dong Zhang, Qianguang Han, Zijie Wang, Zhengkai Huang, Hao Chen, Li Sun, Shuang Fei, Jun Tao, Zhijian Han, Ruoyun Tan, Min Gu, Xiaobing Ju

**Affiliations:** ^1^ Department of Urology, The First Affiliated Hospital of Nanjing Medical University, Nanjing, China; ^2^ Department of Urology, The Second Affiliated Hospital of Nanjing Medical University, Nanjing, China

**Keywords:** interstitial fibrosis and tubular atrophy, kidney transplantation, machine learning algorithms, fibrosis, diagnosis model

## Abstract

**Background:** Interstitial fibrosis and tubular atrophy (IFTA) are the histopathological manifestations of chronic kidney disease (CKD) and one of the causes of long-term renal loss in transplanted kidneys. Necroptosis as a type of programmed death plays an important role in the development of IFTA, and in the late functional decline and even loss of grafts. In this study, 13 machine learning algorithms were used to construct IFTA diagnostic models based on necroptosis-related genes.

**Methods:** We screened all 162 “kidney transplant”–related cohorts in the GEO database and obtained five data sets (training sets: GSE98320 and GSE76882, validation sets: GSE22459 and GSE53605, and survival set: GSE21374). The training set was constructed after removing batch effects of GSE98320 and GSE76882 by using the SVA package. The differentially expressed gene (DEG) analysis was used to identify necroptosis-related DEGs. A total of 13 machine learning algorithms—LASSO, Ridge, Enet, Stepglm, SVM, glmboost, LDA, plsRglm, random forest, GBM, XGBoost, Naive Bayes, and ANNs—were used to construct 114 IFTA diagnostic models, and the optimal models were screened by the AUC values. Post-transplantation patients were then grouped using consensus clustering, and the different subgroups were further explored using PCA, Kaplan–Meier (KM) survival analysis, functional enrichment analysis, CIBERSOFT, and single-sample Gene Set Enrichment Analysis.

**Results:** A total of 55 necroptosis-related DEGs were identified by taking the intersection of the DEGs and necroptosis-related gene sets. Stepglm[both]+RF is the optimal model with an average AUC of 0.822. A total of four molecular subgroups of renal transplantation patients were obtained by clustering, and significant upregulation of fibrosis-related pathways and upregulation of immune response–related pathways were found in the C4 group, which had poor prognosis.

**Conclusion:** Based on the combination of the 13 machine learning algorithms, we developed 114 IFTA classification models. Furthermore, we tested the top model using two independent data sets from GEO.

## Introduction

Among 100,800 solid organ transplants worldwide, 69,400 (62.5%) were renal transplants (Ruenroengbun et al., 2021), which were the most efficacious therapies for patients with uremia due to a variety of causes (Zheng et al., 2022). The incidence of survival of patients with transplanted kidneys has improved with the development of post-transplantation management, but 40% of grafts fail within 10 years of transplantation (Lai et al., 2021). According to traditional theory, allogeneic immune responses cause irreversible kidney damage, resulting in graft loss (Stegall et al., 2011; Shiu et al., 2020). The dominating histopathological findings in the end-stages of renal transplantation include interstitial fibrosis and tubular atrophy (IFTA) and glomerulosclerosis (Djudjaj and Boor, 2019), which are the major cause of graft loss due to progressive loss of function (Franquesa et al., 2012). To determine the prognosis of transplanted kidneys, pathogenesis and prediction models of IFTA are essential to understand.

Over the past few years, the technological advancements of gene sequencing and statistical analysis on a large scale have been applied and developed in genetic diagnosis and analysis. These advancements have enabled the studying of rejection after kidney transplantation to a greater depth. With the immunogenetic approach to kidney transplantation, Dou et al. (2022) developed a prognostic model, which is a good predictor of kidney graft survival at 1 and 3 years. Zhang et al. (2021) discovered two novel prognostic genes associated with ischemia-reperfusion injury, using a total of 1,000 specimens which were collected from 11 independent cohorts. Gu et al. (2021) discovered that renal graft fibrosis, which results in chronic renal graft dysfunction, is negatively regulated by ATG16L.

Necroptosis as a programmed necrosis initiated by agonists such as TNF-α, FasL, and TRAIL was first identified in the field of organ transplantation as being closely related to the quality of the transplanted kidney (Wu et al., 2012; Belavgeni et al., 2020). In addition, in subsequent studies, necroptosis was gradually found to be an important factor involved in or even directly contributing to transplant kidney injury. Linkermann et al. (2012) found that Rip1-induced necrotic apoptosis of renal tubular epithelial cells occurs in early ischemia/reperfusion injury in transplanted kidneys. With the development of necrotic apoptosis, a large number of renal tubular cells undergo acute necrosis, and the renal function further declines, culminating in acute kidney failure (AKI) of the transplanted kidney (Linkermann, 2016). The process of necroptosis releases large amounts of endogenous factors that further promote inflammation and even lead to a prolonged adaptive immune response (Kaczmarek et al., 2013). Both acute necrosis of the renal tubules in the early stages and long-term chronic inflammatory response lead to irreversible damage to the grafts, which in turn lead to fibrosis, a non-specific healing response (Romagnani et al., 2017). Therefore, this study attempted to construct a diagnostic model for IFTA, starting with necrotic apoptosis, an important factor in fibrosis.

In a previous study of IFTA diagnosis based on mRNA expression, the quantity of samples and method of filtering variables were limited (Yang et al., 2022). With the progress of machine learning algorithms, early linear models such as Least Absolute Shrinkage and Selection Operator (LASSO), ridge regression (Ridge), elastic net (Enet), stepwise generalized linear models (Stepglm), linear discriminant analysis (LDA), partial least squares regression (plsRglm), and Naive Bayes have gradually evolved to non-linear models such as support vector machines (SVM), generalized boosted models (glmboost), random forest, gradient boosting machines (GBM), Extreme Gradient Boosting (XGBoost), and artificial neural networks (ANNs) and there are now many options available for the construction of disease diagnostic models. However, on how to construct a low-dimensional diagnostic model based on high-dimensional data that still performs stably in the validation set remains a research challenge. The key benefits of random forest (RF) are its accuracy and resistance to overfitting, which makes it a good choice of machine learning algorithms (Wu et al., 2022), and it has also shown consistent diagnostic efficacy in previous studies (Sun et al., 2022; Wu et al., 2022; Xiang et al., 2022).

The purpose of this study is to construct 114 algorithm combinations based on LASSO, Ridge, Enet, Stepglm, SVM, glmboost, LDA, plsRglm, random forest, GBM, XGBoost, Naive Bayes, and ANNs and finally to screen out the diagnostic model with the best results by comparing the average AUC of each model in the training group and test group. We then used GSE21374 to further explore the influence of genes used to construct a diagnostic model of IFTA on long-term graft loss.

## Materials and methods

### Data retrieval and organization

We performed a comprehensive search on the GEO official website for expression matrix and annotated clinical information of patients who underwent transplantation of the kidney. The screening criteria for 162 “kidney transplant” cohorts in the GEO were 1) all probes in each sample have a value greater than 0; 2) each patient’s sample with publicly available gene expression profile contained biopsy-confirmed IFTA information or survival information about long-term graft loss; and 3) The total number of cohort samples is ≥50. Finally, we selected five data sets (GSE22459, GSE53605, GSE76882, GSE98320, and GSE21374) to be included in this study (Park et al., 2010; Modena et al., 2016; Bontha et al., 2017; Reeve et al., 2017). As part of the data processing, the original data matrix was downloaded, probe annotation performed, and low-abundance genes in most samples were removed. Each data set contained samples classified as IFTA and non-IFTA, and the detailed description about the groups is shown in Table 1. We used the combat function in the SVA package to remove the batch effects of GSE76882 and GSE98320 and construct the training set, and evaluate the efficacy of the model using GSE22459 and GSE53605 as the test sets, respectively.

**TABLE 1:** Details of GEO data sets that meet the filter criteria.

GEO no	Platform	Species	Tissues	Non-IFTA	IFTA	Total	Group
GSE22459	GPL570	*Homo sapiens*	Kidney biopsy	25	40	65	Training set
GSE53605	GPL571	*Homo sapiens*	Kidney biopsy	45	10	55	Training set
GSE76882	GPL13158	*Homo sapiens*	Kidney biopsy	139	135	274	Validation set
GSE98320	GPL15207	*Homo sapiens*	Kidney biopsy	974	234	1208	Validation set
GSE21374	GPL570	*Homo sapiens*	Kidney biopsy			282	Prognosis set

### Construction of gene sets for algorithms of machine learning

Using “necroptosis” as a keyword in GeneCards (https://www.genecards.org/), the resulting genes were filtered according to the following criteria: 1) they are protein coding genes 2) the correlation score with “necroptosis” is more than 0.8. Through the “limma” package in R (version 4.1.1) (Ritchie et al., 2015), we extracted the differentially expressed genes (DEGs) in the training set, with adjusted *p*-values = 0.05 as the filter criteria; then, by taking the intersection of the DEGs and gene set of necroptosis, the DEGs were visualized using the “ggplot2” R packages.

### Building and evaluating models

The models were first established using 13 machine learning methods, and then further downscaling of the generated models was carried out by combining the different modeling methods. In the end, we constructed 114 diagnostic models for IFTA. The AUC values of the generated models in the training set and test sets were calculated. Finally, the models were ranked according to the average AUC values to filter the optimal models. The model efficacy was then further visualized by plotting the ROC and using the principal component analysis (PCA).

### Unsupervised clustering of kidney transplantation patients

In order to identify potential molecular subtypes of transplanted kidneys based on 28 key biomarkers, an unsupervised cluster analysis was performed on the prognosis set using the “ConsensusClusterPlus” R package, with the resampling set as 1,000. Classification stability was ensured by using the k-means algorithm with 1,000 iterations and an 80% resampling rate. The PCA maps are then plotted using the t-distributed stochastic neighbor embedding (tSNE) algorithm to further visualize the differences between the clusters. A Kaplan–Meier (KM) survival curve was used to determine the survival differences among the subtypes of kidney transplantation patients. The best and worst prognostic groups were analyzed for differences with the other groups using the limma package, and the DEGs were extracted.

### Functional enrichment analysis

To further explore the source of the prognostic differences between the different subgroups, we first transformed the gene expression matrices of the prognosis sets into hallmark enrichment matrices using the GSVA package, and visualized the results using the pheatmap package. To further explore the reasons for the two groups with the largest prognostic differences, we performed Gene Ontology (GO) enrichment analysis on the genes of the upregulated groups obtained from the two difference analyses, using the “clusterProfiler” R package (Yu et al., 2012). CIBERSORT, a deconvolution algorithm that quantifies cell types based on the gene matrix, was used to quantify 22 kinds of immune cell infiltration, and then a map of immune cell infiltration was drawn to determine the difference of four subtypes by unsupervised clustering, using the R package “ggpubr”. To further validate the results of CIBERSORT, we similarly used enrichment analyses of immune function by the ssGSEA for the four subgroups.

## Results

### Data organization and screening of genes for modeling

**FIGURE 1:**
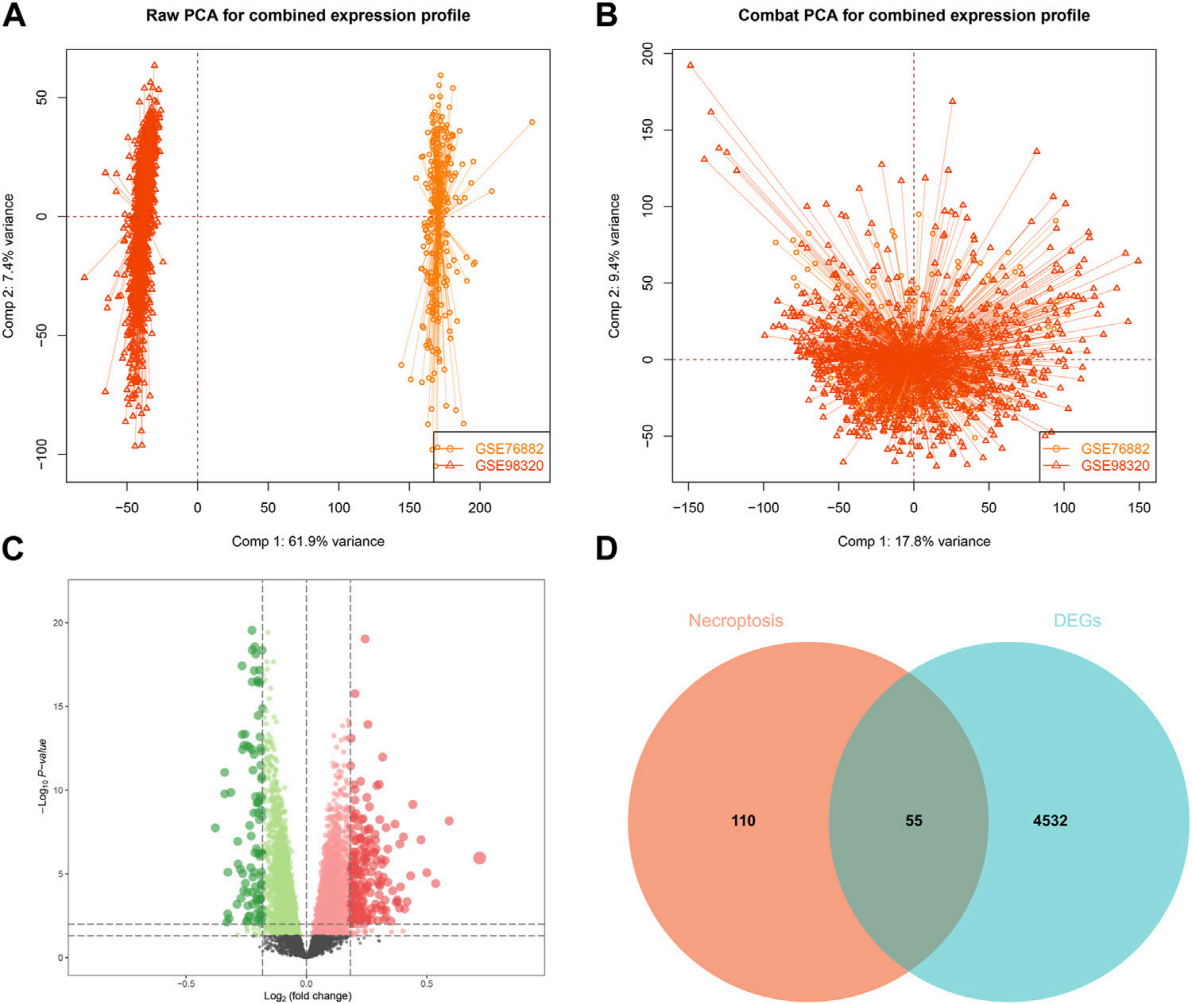
Construction and differential gene expression of the training set. **(A)** GSE98320 and GSE76882 before de-batching. **(B)** GSE98320 and GSE76882 after de-batching. **(C)** Volcanic maps of training set. **(D)** Extraction of necroptosis-related differential genes.

Since the data sets used for modeling and validation were sequenced using different platforms, we first took the intersection of the four data set genes and ended up with 10,721 shared genes. GSE76882 and GSE98320 used for modeling were then de-batched by the combat function, and the change in the PCA plots from Figures 1A, B shows that the batch effect had been eliminated for both. Using the “limma” R package, a total of 4,587 DEGs were identified according to the training set. There was a significant difference between the IFTA and non-IFTA groups in these genes, as shown in Figure 1C. The detailed results of the differential analysis can be found in Supplementary Table S1. A total of 165 necroptosis-related genes were obtained by filtering the results obtained from the GeneCard database search (Supplementary Table S2). By intersecting these two gene sets, a total of 55 necroptosis (Figure 1D) with differences between IFTA and non-IFTA groups of the training set were obtained (Supplementary Table S3).

### Building and evaluating models

**FIGURE 2:**
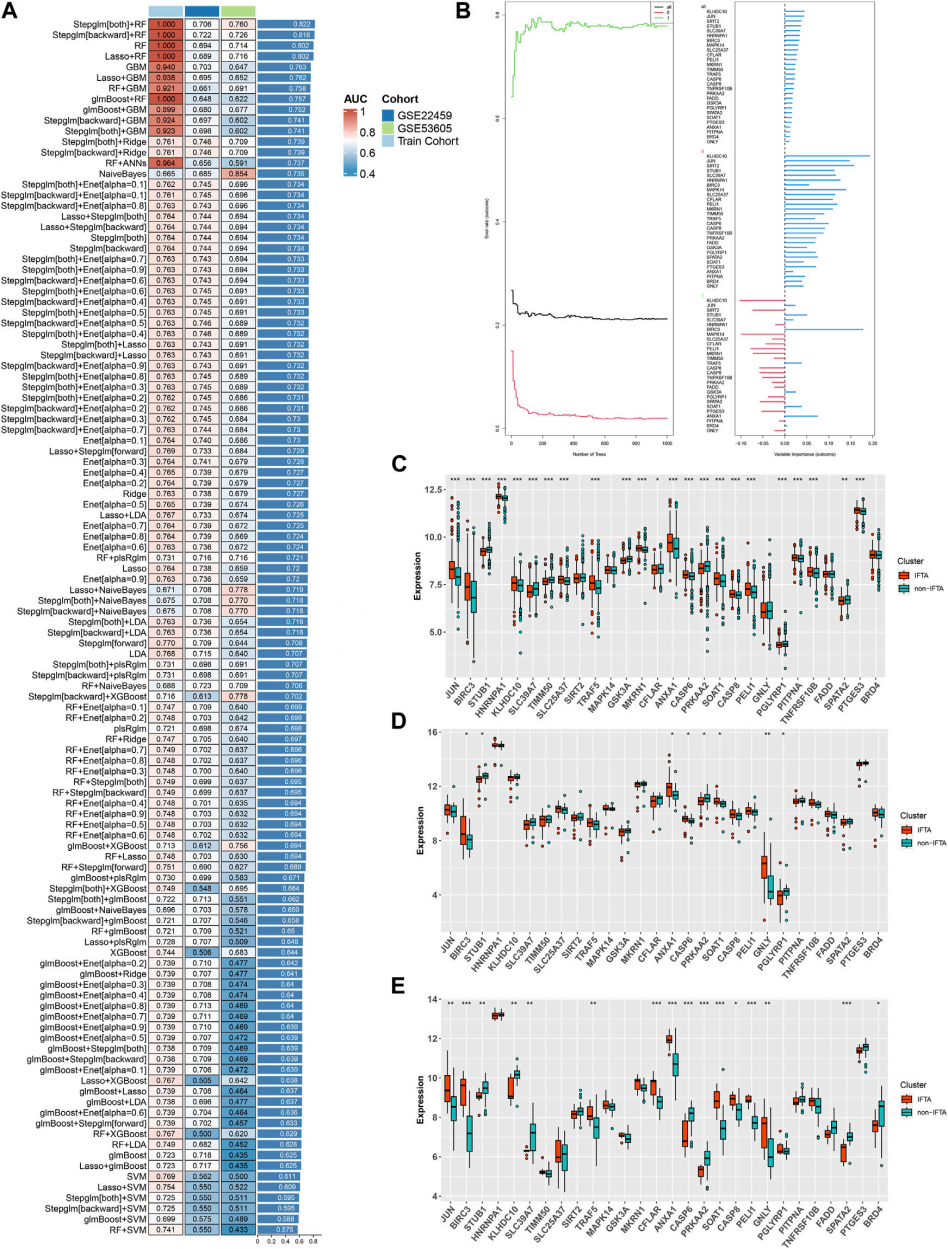
Construction and validation of the IFTA diagnostic model by machine learning algorithm. **(A)** Screening the optimal model based on the mean AUC of 114 diagnostic models in the modeling and validation groups. **(B)** Visualization of the random forest model constructed from 28 genes based on Stepglm[both] screening. **(C–E)** Differential expression of modeling genes in the non-IFTA and IFTA groups; *p < 0.05, **p < 0.01, ***p < 0.001. **(C)** Training set. **(D)** GSE22459. **(E)** GSE53605.

**FIGURE 3:**
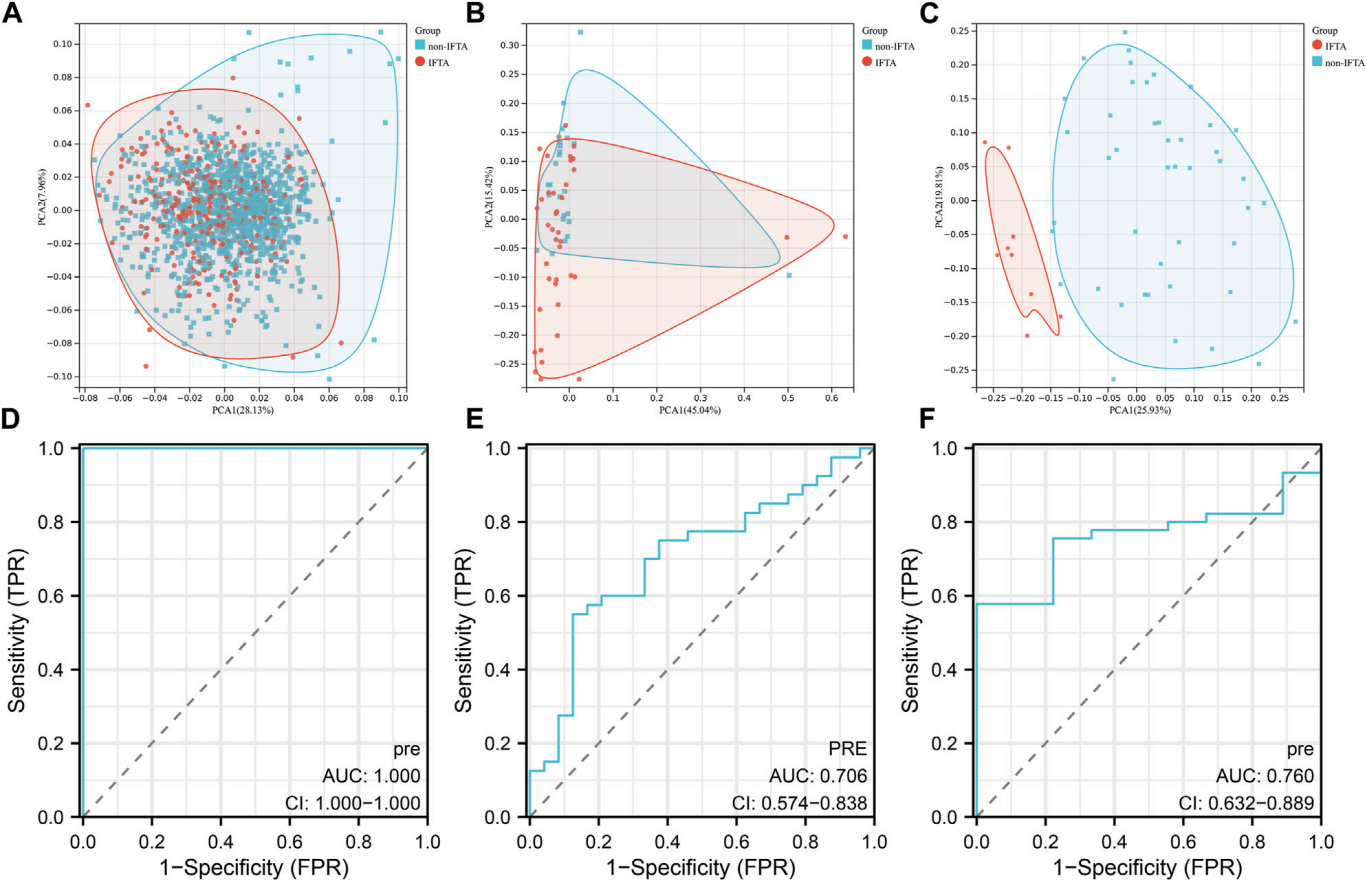
Validation of the optimal diagnostic model. **(A–C)** Principal component analysis (PCA) of training set **(A)**, GSE22459 **(B)**, GSE53605 **(C)**. **(D–F)** ROC curves of training set **(D)**, GSE22459 **(E)**, and GSE53605 **(F)**.

Based on the 55 necroptosis-related genes with differential expression obtained from the previous screening, we used LASSO, Ridge, Enet, Stepglm, SVM, glmboost, LDA, plsRglm, random forest, GBM, XGBoost, Naive Bayes, and ANNs to construct 114 diagnostic models. By visualizing the AUC values of the 114 models (Figure 2A), it can be seen that Stepglm[both]+RF is one of the 114 diagnostic models that shows good predictive power in the training group and validation groups. Twenty-eight genes with a high correlation with IFTA were screened by Stepglm[both] (Table 2). A non-linear model was constructed based on these 28 genes using the rfsrc function in the randomForestSRC package (Figure 2B). Visualizing the differences in the expression of these 28 genes between the IFTA and non-IFTA groups in the training group and the other two validation sets (Figures 2C–E). Figures 3A–C show that there is a distinction between the IFTA group and non-IFTA group. The AUC values of the training set and validation cohorts are 1.000, 0.706, and 0.760 (Figures 3D–F).

**TABLE 2:** Necroptosis-related genes strongly associated with IFTA screened by Stepglm[both].

Vars	Coef.	Odds ratio	Coef. 95% CI
ANXA1	0.351	1.42	0.094 to 0.609
BIRC3	0.725	2.065	0.456 to 1.000
BRD4	0.171	1.186	0.011 to 0.334
CASP6	0.145	1.155	−0.054 to 0.344
CASP8	0.195	1.215	0.019 to 0.372
CFLAR	−0.271	0.762	−0.436 to −0.108
FADD	0.244	1.276	0.044 to 0.445
GNLY	−0.173	0.841	−0.34 to −0.008
GSK3A	−0.254	0.775	−0.442 to −0.069
HNRNPA1	0.381	1.464	0.162 to 0.604
JUN	0.33	1.391	0.161 to 0.499
KLHDC10	0.314	1.37	0.166 to 0.467
MAPK14	−0.321	0.726	−0.551 to −0.094
MKRN1	0.174	1.19	−0.023 to 0.372
PELI1	−0.197	0.821	−0.433 to 0.039
PGLYRP1	−0.126	0.882	−0.28 to 0.026
PITPNA	0.216	1.241	0.025 to 0.41
PRKAA2	−0.325	0.722	−0.515 to −0.134
PTGES3	−0.277	0.758	−0.521 to −0.035
SIRT2	0.334	1.396	0.153 to 0.517
SLC25A37	0.166	1.181	0.005 to 0.33
SLC39A7	−0.382	0.682	−0.582 to −0.185
SOAT1	−0.369	0.691	−0.618 to −0.125
SPATA2	0.138	1.148	−0.028 to 0.306
STUB1	−0.32	0.726	−0.527 to −0.116
TIMM50	−0.182	0.833	−0.378 to 0.012
TNFRSF10B	−0.34	0.712	−0.576 to −0.106
TRAF5	−0.438	0.645	−0.716 to −0.163

### Subgroup analysis of IFTA

**FIGURE 4:**
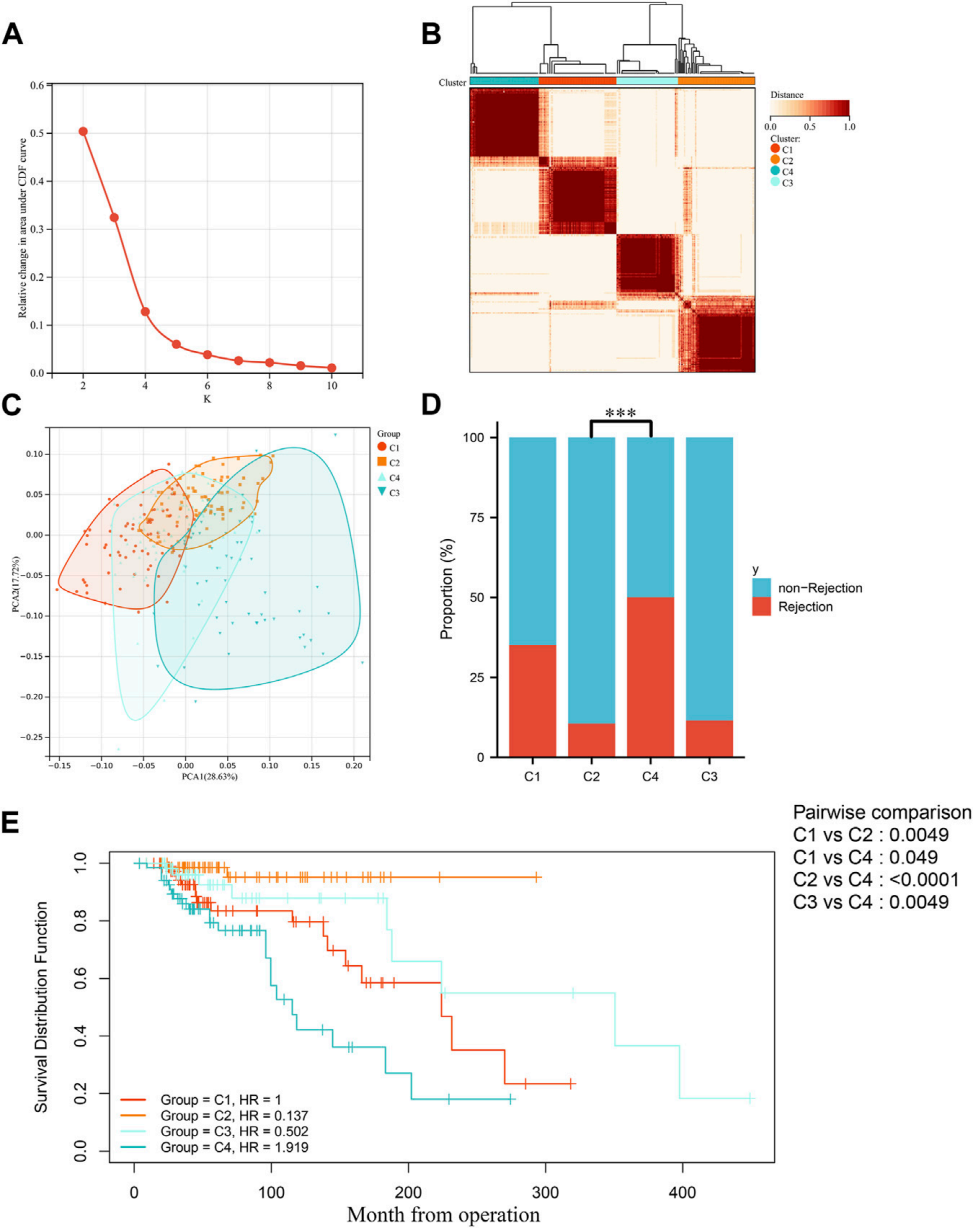
Identification of molecular subgroups in IFTA. **(A)** Relative variation of the area under the CDF region at k = 2–9. **(B)** Consensus clustering matrix when k = 4. **(C)** PCA of four clustered groups. **(D)** Comparison of the occurrence of acute rejection of grafts in four subgroups. **(E)** Comparison of KM survival curves for post-transplantation graft loss occurring between four subgroups.

In order to explore the roles of biomarkers in kidney transplantation prognosis, the prognosis sets were classified using consensus clustering. The number of iterations was set at 1,000 times to ensure stability of the classification categories. The results suggest that when the number of clusters (k) was 4, the samples in the consensus profiles obtained an optimal allocation (Figures 4A, B). Furthermore, by PCA, it can be seen that there are very significant differences between the four subgroups (Figure 4C). The KM survival curves were then plotted based on the four subgroups, which showed that the C2 group had the best prognosis, with the lowest percentage of kidney graft loss occurring as time progressed post transplantation, in contrast to C4, which had the worst prognosis (Figure 4E). As seen in Figure 4D, by comparing the proportion of AR (Acute Rejection) occurring in the C2 and C4 groups, where the difference in survival is most pronounced, AR accounts for a significantly higher proportion of the C4 group, which has a poorer prognosis and is indicative of the fact that necroptosis may play a contributory role in the development and progression of AR.

**FIGURE 5:**
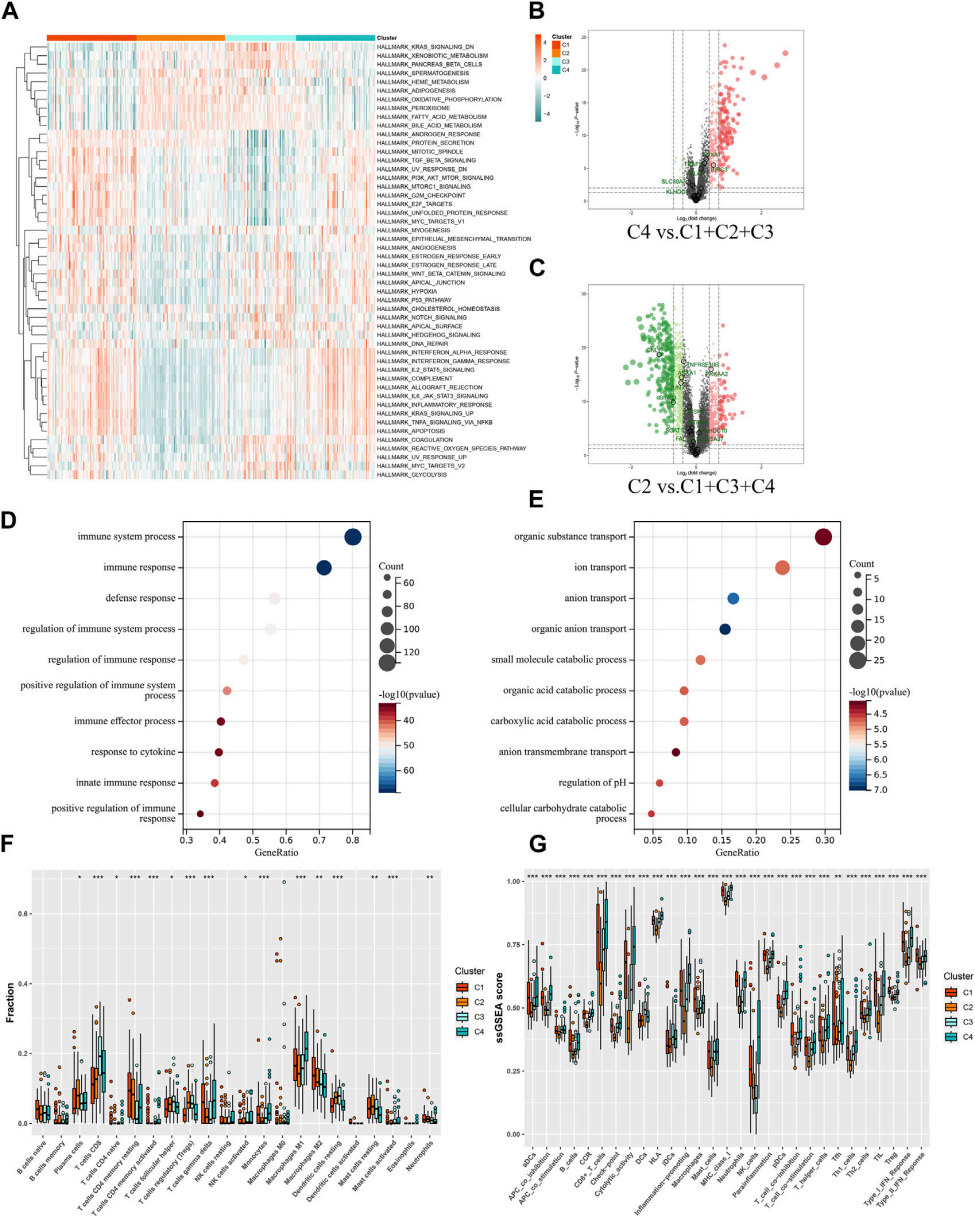
Functional analysis of molecular subgroups in IFTA. **(A)** Heatmap of four clusters based on Z-scores of ssGSEA of 50 hallmarks. **(B, C)** Volcano plots for differentially expressed genes (FDR > 0.05 |FC| > 1.5) in different subgroups. **(B)** C4 vs. C1 + C2 + C3. **(C)** C2 vs. C1 + C3 + C4. **(D, E)** GO enrichment analysis of C2 and C4 upregulated genes. **(D)** C4. **(E)** C2. **(F)** Differences in infiltrated immune cells by CIBERSORT; *p < 0.05, **p < 0.01, ***p < 0.001. **(G)** Differences of immune function by ssGSEA; *p < 0.05, **p < 0.01, ***p < 0.001.

By enrichment analysis of the hallmark of the four groups, it is seen that the difference between the C2 and C4 groups is extremely obvious, with C2 having significant upregulation in the KRAS signaling pathway–downregulated gene set, oxidative phosphorylation gene set, and xenobiotic metabolism gene set, whereas the C4 group, which has poorer prognosis, has significant upregulation in the gene set for allogeneic transplantation rejection, inflammatory response gene set, apoptosis gene set, TGF-β signal pathway gene set, and angiogenesis gene set (Figure 5A). To further validate the aforementioned enrichment analysis results, we extracted the upregulated genes from the C2 and C4 groups, using FDR > 0.05 |FC| > 1.5 as the screening criterion (Figures 5B, C). The GO enrichment analysis of the set of upregulated genes showed similar results to those of the hallmark enrichment analysis, that is, the upregulated biological processes in the C4 group were mainly in the immune-related processes (Figure 5D), whereas there was a significant upregulation of various substance metabolisms mainly in the C2 group (Figure 5E). We then used CIBERSORT to try and further explore the differences in immune cell infiltration between the different subgroups. Screening the samples at *p* < 0.05 (Figure 5F) showed that the most significant subgroups of differences in the four groups were mainly in the T cells of the thymic and macrophage lineages. To further validate the results of the CIBERSORT immune cell infiltration analysis, we again used ssGSEA to enrich different immune functions, and it was seen that all of the immune function scores were significantly different (Figure 5G).

## Discussion

IFTA is not only the commonly occurring histopathological manifestation of chronic kidney disease (CKD) but also the cause of long-term renal failure in transplanted kidneys (Franquesa et al., 2012; Djudjaj and Boor, 2019). As a result of chronic fibrosis of the transplanted kidney, IFTA develops in the early stages after transplantation, eventually causing renal failure (Brouard et al., 2011). Although the diagnostic model for IFTA can clearly identify it at the early stages and is helpful in judging the prognosis of transplanted kidneys, the detection method of the process is rare. The more popularly accepted view of the onset and progression of fibrosis is that the graft receives irreversible damage, ensures its basic structural stability, and is repaired by a non-specific healing response like interstitial fibrosis (Romagnani et al., 2017). There are many reasons for irreversible damage to grafts; necroptosis being one of them induces an adaptive immune system that can lead to chronic fibrosis and even later on to a massive loss of nephrons leading to renal decompensation (Maremonti et al., 2022). Calcineurin inhibitors (CNIs), that are heavily used in renal transplantation patients, also cause changes in the morphological characteristics of renal tubules through necroptosis (Claus et al., 2018). Therefore, in this study, we started by exploring necroptosis, one of the causes of IFTA, with 55 necroptosis-related genes with differential expression, applied 13 machine learning methods to analyze the target data set in the GEO database, established 114 IFTA diagnostic models, and compared the AUC values of the models to arrive at the optimal model.

The best performing of our models of 114 machine learning models was Stepglm[both]+RF, with an average AUC value of 0.822 in the one modeling and two validation groups. The top 4 out of the 114 machine learning methods were all non-linear models constructed by RF, which shows that RF is indeed superior to the other methods in preventing model overfitting. RF can automatically explore the interactions among multiple variables and find the variables that matter more when dealing with high-dimensional data. Furthermore, RF can avoid overfitting by generating a large number of decision trees at random. As a result, RF has demonstrated satisfactory accuracy both in the modeling and validation groups in previous studies (Zhong et al., 2018).

The performance of the model in the validation group GSE22459 was mediocre, with an AUC value of only 0.706, and the results of the PCA also indicated that IFTA did not have high discriminatory power. By contrast, the model performed much better in GSE53605, with an AUC value of 0.760, and the results of the PCA also indicated that there was a clear distinction between the IFTA and non-IFTA groups. By visualizing the differences in the expression of the modeled genes, we conclude that the 28 genes used for modeling show significant differences in both the training group and GSE53605, while the differences between the two groups visible in GSE22459 are not significant. From this, we hypothesize that the reason for the model’s mediocre performance in GSE22459 is that first, the expression matrices of both the modeling and validation sets are derived from sequencing files from different platforms and there is a large batch effect between the gene sets, and second, the sample size of the validation set is very small when compared to that of the training set.

To further explore the role of the modeling genes in the distant future of transplanted kidneys, we performed unsupervised clustering of the prognosis set based on the expression of 28 modeling genes, and finally obtained four subgroups, in which the C2 group had the last prognosis and the C4 group had the worst prognosis. In addition, in the subsequent enrichment analysis, it was found that the poorer prognosis C1 and C4 groups were enriched, having significant upregulation of the TGF-β signal pathway—the graft rejection pathway associated with fibrosis. It can be seen that the C4 group obtained by unsupervised clustering with the IFTA diagnostic model is the group with the highest degree of fibrosis and the worst prognosis, which can be used in future to classify the risk of patients in the early stages of transplantation, so that high-risk patients can be followed up with high-frequency screening and prevent the occurrence of post-transplantation adverse events or even the loss of transplanted kidneys.

Even so, several limitations also exist in this study. First, transplanted kidney biopsy tissue samples were used as model input data, and acquiring tissues is challenging in clinical practice. Second, further experimental studies are required to explain the correlation between certain biomarkers and IFTA mechanistically. However, too many genes were used in constructing the model, and in considering that other models with a much smaller number of genes performed poorly in both the modeling and validation groups, we ended up choosing the diagnostic model constructed by Stepglm[both]+RF. Third, further exploration of clusters C4 and C2 was hampered by a lack of clinical information. Finally, we screened the entire transplant kidney cohorts in the GEO database to build a diagnostic model. However, to build models, machine learning algorithms require a larger number of samples. More independent kidney transplantation patient cohorts should be used to evaluate and elevate our model’s performance. In order to verify patient tissue samples, we will have to collect samples from our hospital.

## Conclusion

In summary, based on the combination of 13 machine learning algorithms, we developed 114 IFTA classification models. Furthermore, we tested the top model using two independent data sets from GEO.

## Data Availability

The datasets presented in this study can be found in online repositories. The names of the repository/repositories and accession number(s) can be found in the article/Supplementary Material.
