# Supplementary figures and images for: Construction of predictive model of interstitial fibrosis and tubular atrophy after kidney transplantation with machine learning algorithms
Source: Front Genet. 2023 Nov 1;14:1276963. doi: 10.3389/fgene.2023.1276963 (PMC10646529; doi:10.3389/fgene.2023.1276963)

Survival Distribution Function

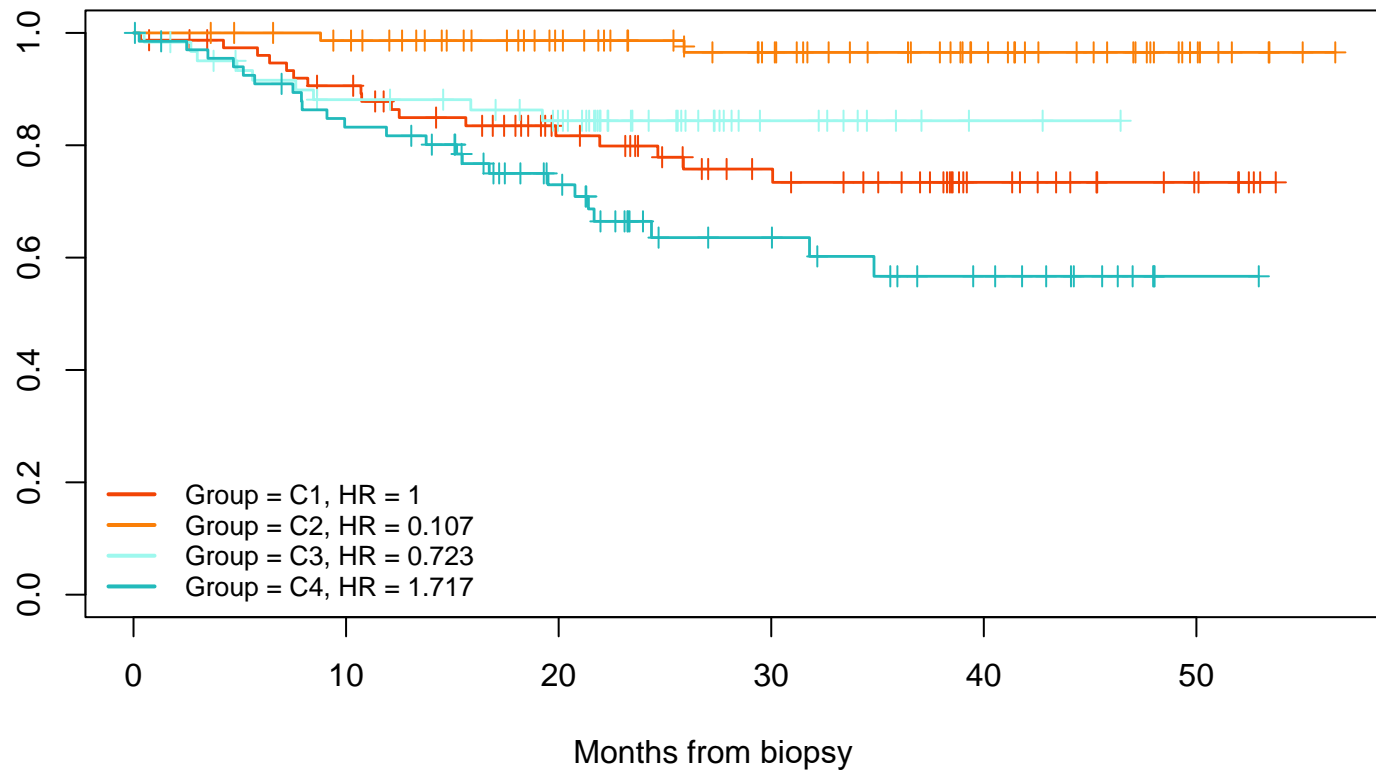

Pairwise comparison

C1 vs C2 :  $7e-04$

C2 vs C3 : 0.0131

C2 vs C4 :  $<0.0001$

C3 vs C4 : 0.0369

Supplement: Supplementary file 2 [file Image1.pdf]
